# Geospatial and temporal associations of Getah virus circulation among pigs and horses around the perimeter of outbreaks in Japanese racehorses in 2014 and 2015

**DOI:** 10.1186/s12917-017-1112-6

**Published:** 2017-06-19

**Authors:** Hiroshi Bannai, Manabu Nemoto, Hidekazu Niwa, Satoshi Murakami, Koji Tsujimura, Takashi Yamanaka, Takashi Kondo

**Affiliations:** 1 Equine Research Institute, Japan Racing Association, 1400-4 Shiba, Shimotsuke, Tochigi 329-0412 Japan; 2 Thermo Fisher Scientific, Life Technologies Japan Ltd, 4-2-8 Shibaura, Minato-ku, Tokyo, Japan

**Keywords:** Getah virus, Japan, Horses, Pigs, Virus circulation

## Abstract

**Background:**

We studied a recent epizootic of Getah virus infection among pigs in the southern part of Ibaraki Prefecture and the northern part of Chiba Prefecture, Japan, focusing on its possible association with outbreaks in racehorses in 2014 and 2015. The genomic sequence of a Getah virus strain from an infected pig was analyzed to evaluate the degree of identity with the strains from horses.

**Results:**

Sera were collected from pigs from September to December 2012 to 2015 in south Ibaraki (380 pigs in 29 batches), and from September to December 2010 to 2015 in north Chiba (538 pigs in 104 batches). They were examined by using a virus-neutralizing test for Getah virus. Seropositivity rates in 2012–2013 in south Ibaraki and 2010–2012 in north Chiba ranged from 0% to 1.6%. In south Ibaraki, seropositivity rates in 2014 (28.8%) and 2015 (65.0%) were significantly higher than those in the previous years (*P* < 0.01); 4/5 batches had positive sera in 2014 and 7/7 in 2015. In north Chiba, seropositivity rates in 2013 (14.1%), 2014 (17.8%), and 2015 (48.0%) were significantly higher than those in the previous years (*P* < 0.01); 6/27 batches had positive sera in 2013, 3/9 in 2014, and 5/5 in 2015. Complete genome analysis revealed that the virus isolated from an infected pig had 99.89% to 99.94% nucleotide identity to the strains isolated from horses during the outbreaks in 2014 and 2015.

**Conclusions:**

Serological surveillance of Getah virus in pigs revealed that the virus was circulating in south Ibaraki and north Chiba in 2014 and 2015; this was concomitant with the outbreaks in racehorses. The Getah virus strain isolated from a pig was closely related to the ones from horses during the 2014 and 2015 outbreaks. To our knowledge, this is the first convincing case of simultaneous circulation of Getah virus both among pigs and horses in specific areas.

## Background

Getah virus is classified in the genus *Alphavirus* in the family *Togaviridae* [1]. It is mosquito-borne and is widespread from Eurasia to Australasia. This virus causes fever, skin eruptions, and limb edema in horses [1], and it causes fetal death and reproductive disorders in pigs [2, 3]. Both pigs and horses can play important roles in the amplification and circulation of Getah virus, because maximum virus titers in the blood of infected animals of these species are sufficient for the vector mosquitoes to be infected, although viremia does not last for a long period [1, 4–6]. *Aedes vaxans nipponii* and *Culex tritaeniorhynchus* are considered to be major species that transmit Getah virus in Japan [7].

**Fig. 1:**
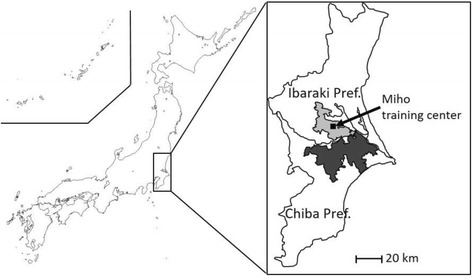
Locations of the study areas. The area in the southern part of Ibaraki Prefecture (south Ibaraki, gray) includes the village of Miho and the cities of Inashiki, Ushiku, and Tsuchiura and covers an area of 454 km^2^. There are 27 pig farms rearing about 32,000 pigs. The area in the northern part of Chiba Prefecture (north Chiba, black) includes the towns of Tako and Tohnosho and the cities of Inzai, Katori, Sakura, Narita, Shiroi, Yachimata, and Tomisato and covers an area of 988 km^2^. There are 158 pig farms rearing about 230,000 pigs. The location of the Miho training center—the site of the outbreak of Getah virus in racehorses in 2014 and 2015—is indicated by the black square

Outbreaks of Getah virus infection in horses occurred in autumn 2014 and 2015 at the Miho training center of the Japan Racing Association after 31 years of no reported outbreaks in Japan [8, 9]. The Miho training center is located in Ibaraki Prefecture in eastern Japan (Fig. 1). Surrounding the center, in Ibaraki and the neighboring Chiba Prefecture, are more than 30 private racehorse farms. Our previous study revealed that the virus was epizootic not only at the training center in 2014 and 2015, but also at the private horse farms surrounding the center [9, 10]. Because these two prefectures are major producers of pigs, the involvement of pigs in Getah virus circulation in the area and their association with outbreaks in horses have been suspected. However, there have been only a few recent reports on the prevalence of Getah virus among pigs. Serological surveillance in 2010–2014 revealed continuous circulation of the virus among pigs in the Kanto region, which includes Ibaraki and Chiba prefectures [*Reproduction Disorders Caused by Getah Virus Infection* (Kyoto Biken Information for Swine Veterinarians, 2013, no. 5) and *Prevention of Pigs from Getah Virus Infection* (Kyoto Biken Information for Swine Veterinarians, 2015, no. 10), in Japanese; http://kyotobiken.sakura.ne.jp/security/index.php]. However, detailed information on sample collection, such as numbers of farms, numbers of pigs, and time periods, were not indicated in these studies, and it is not clear whether the magnitude of the epizootic outbreaks was larger in 2014 and 2015 than in the previous years. Hence, the association of pigs with the Getah virus outbreaks in horses was only speculative.

Here, we investigated Getah virus seropositivity among pigs in the southern part of Ibaraki Prefecture and the northern part of Chiba Prefecture, around the Miho training center, in the period 2010–2015, focusing on a possible association with the outbreaks in racehorses in 2014 and 2015. To further clarify the interactive circulation of the virus between the species, we isolated a Getah virus strain from a pig and compared its genomic sequence with those of previous equine isolates.

## Methods

### Study areas and pig sera

The study area in the southern part of Ibaraki Prefecture (south Ibaraki) included the village of Miho and the cities of Inashiki, Ushiku, and Tsuchiura, covering an area of 454 km^2^; there are 27 pig farms rearing about 32,000 pigs (Fig. 1). The area in the northern part of Chiba Prefecture (north Chiba) included the towns of Tako and Tohnosho and the cities of Inzai, Katori, Sakura, Narita, Shiroi, Yachimata, and Tomisato, covering an area of 988 km^2^; there are 158 pig farms rearing about 230,000 pigs. Sera were collected from pigs at various times from September to December 2012 to 2015 in south Ibaraki (380 pigs in 29 batches) and from September to December 2010 to 2015 in north Chiba (538 pigs in 104 batches). They were kindly provided by South Ibaraki Livestock Hygiene Service Center and Chiba Central Livestock Hygiene Service Center. Each batch represented 2 to 52 serum samples (mean, 6.5 samples) collected at one farm on one day. Some batches were collected at multiple time points on the same farms, but not in the same years. In this case, the samples collected at different time points were regarded as different batches, because they should reflect different virus-exposure status. The numbers of batches and serum samples collected in each month are shown in Tables 1 and 2. The pigs were 4 to 6 months old and had not been vaccinated with Getah virus vaccine. Also, the maternal antibodies against most pathogens were considered to be undetectable in 4–6 months old piglets. Therefore, any antibodies present should be the result of virus exposure within the previous 4 to 6 months.

**Table 1:** Rates of seropositivity to Getah virus among pigs in south Ibaraki in autumn 2012–2015

Year		Sep.	Oct.	Nov.	Dec.	Total
2012	No. samples	0/17 (0%)^a^	-	1/47 (2.1%)	0/36 (0%)	1/100 (1.0%)
	No. batches	0/2	-	1/4	0/2	1/8
2013	No. samples	0/25 (0%)	0/41 (0%)	0/26 (0%)	0/5 (0%)	0/97 (0%)
	No. batches	0/2	0/4	0/2	0/1	0/9
2014	No. samples	6/30 (20.0%)	12/21 (57.1%)	1/15 (6.7%)	-	19/66 (28.8%)
	No. batches	1/2	2/2	1/1	-	4/5
2015	No. samples	2/6 (33.3%)	53/83 (63.9%)	20/26 (76.9%)	2/2 (100.0%)	77/117 (65.0%)
	No. batches	1/1	2/2	3/3	1/1	7/7

^a^No. of positive samples/No. of tested samples (positivity rate)

**Table 2:** Rates of seropositivity to Getah virus among pigs in north Chiba in autumn 2010–2015

Year		Sep.	Oct.	Nov.	Dec.	Total
2010	No. samples	2/68 (2.9%)^a^	0/35 (0%)	0/15 (0%)	0/5 (0%)	2/123 (1.6%)
	No. batches	2/14	0/7	0/3	0/1	2/25
2011	No. samples	0/45 (0%)	0/27 (0%)	0/5 (0%)	0/34 (0%)	0/111 (0%)
	No. batches	0/9	0/6	0/1	0/7	0/23
2012	No. samples	0/10 (0%)	0/5 (0%)	0/29 (0%)	0/30 (0%)	0/74 (0%)
	No. batches	0/2	0/1	0/6	0/6	0/15
2013	No. samples	0/60 (0%)	6/40 (15.0%)	9/15 (60.0%)	4/20 (20.0%)	19/135 (14.1%)
	No. batches	0/12	3/8	2/3	1/4	6/27
2014	No. samples	0/10 (0%)	8/35 (22.9%)	-	-	8/45 (17.8%)
	No. batches	0/2	3/7	-	-	3/9
2015	No. samples	-	24/50 (48.0%)	-	-	24/50 (48.0%)
	No. batches	-	5/5	-	-	5/5

^a^No. of positive samples / No. of tested samples (positivity rate)

### Cell culture

For virus-neutralization (VN) testing and virus isolation we used Vero cells (Sumitomo Dainippon Pharma, Tokyo, Japan). Cells were cultured in minimum essential medium (MEM, MP Biomedicals, Irvine, CA, USA) containing 10% fetal calf serum (Sigma Aldrich Inc., St. Louis, MO, USA), 100 units/ml penicillin, and 100 μg/ml streptomycin (Sigma Aldrich Inc.). MEM containing 2% fetal calf serum, 100 units/ml penicillin, and 100 μg/ml streptomycin was used as a maintenance medium for VN testing and virus isolation.

### VN testing for Getah virus

For VN testing, we used Getah virus strain 14-I-605 isolated from a racehorse during the 2014 Getah virus outbreak [10]. Sera were diluted at 1:4 with the maintenance medium and inactivated for 30 min at 56 °C. They were then transferred on flat-bottomed 96-well plates (Asahi Glass Co. Ltd., Tokyo, Japan) in duplicate and mixed with an equal volume of the prepared virus (one hundred 50%-tissue-culture infective doses/50 μl/well). After incubation of the wells for 1 h at 37 °C with 5% CO_2_, Vero cells were added at a concentration of 2.5 × 10^4^ cells/50 μl/well. After incubation for 3 days at 37 °C with 5% CO_2_, the cells were stained with crystal violet – formalin solution. The test was performed with single dilution of 1:4, and the sera were defined as positive for VN antibodies when the cytopathic effect was completely inhibited. Statistical significance for seropositive rates between the years was analyzed by z-test.

### Detection of Getah virus RNA in pig sera

To detect Getah virus RNA efficiently, we used seronegative samples collected from farms suspected to be contaminated with Getah virus. Serum batches that were collected in September and October of each year and that included seropositive samples were tested for Getah virus RNA by using RT-PCR. Five sera from each batch were pooled (20 μl for each sample), and each pool included as many seronegative samples as possible. For some batches that included more than five seronegative samples, multiple pools were tested. If the number of seronegative samples was not enough to make a pool, seropositive samples were selected randomly from the batch. In total, 24 pools were tested. RNA was extracted from pooled sera by using a nucleic acid isolation kit (MagNA Pure LC Total Nucleic Acid Isolation Kit, Roche Diagnostics, Mannheim, Germany), and viral gene detection was performed by using an RT-PCR for the Getah virus *non-structural protein 1 (nsP1)* gene using primer sets M_2_W-S and M_3_W-S [11].

### Virus isolation

In the case of those pooled sera that were positive for Getah virus RNA, 25 μl of original sera that had been used to make the corresponding pool was inoculated simultaneously with Vero cells (5 × 10^5^ cells/2 ml/well) on each well of a six-well plate (Asahi Glass Co. Ltd.). The next day, the cells were washed three times with phosphate-buffered saline and cultured in maintenance medium. To identify Getah virus–specific nucleotide sequences, the supernatants of inoculated cells that showed cytopathic effects were tested by using RT-PCR as described above and sequenced by Filgen Inc. (Nagoya, Japan).

### Complete-genome sequencing of Getah viruses, and phylogenetic analysis

Strain 15-I-752, which was isolated from a horse in 2015 [9], and strain 15-I-1105, which was isolated from a pig in this study, were sequenced. Viral RNA was extracted by using a MagNA Pure LC Total Nucleic Acid Isolation Kit (Roche Diagnostics, Mannheim, Germany). RT-PCR was performed with three primer sets (Getah F1–21 and Getah R5366–5345; Getah F5009–5028 and Getah R8649–8630; and Getah F8401–8424 and Getah R111305–11286), as described previously [12], and was conducted with a PrimeScript II High Fidelity RT-PCR Kit (Takara Bio Inc., Shiga, Japan). PCR amplicons were sequenced by using Ion Torrent technology (Thermo Fisher Scientific, MA, USA). Libraries were constructed by using an Ion Xpress Plus Fragment Library Kit and an Ion Xpress Barcode Adapters Kit. Emulsion PCR, enrichment and loading onto an Ion 316 Chip were performed automatically with Ion Chef, and sequencing was conducted with the Ion PGM system according to the manufacturer’s instructions. The average depth was 6402.5 times. The raw signal data were analyzed by using Torrent Suite version 5.0.5, and 14-I-605-C1 (LC079088) was used as a reference sequence. Torrent Variant Caller version 5.0.4.0 was used to call the mutated sites, and the most frequently observed alleles were selected in each of the called positions. Bases within the reference sequence were substituted with the observed alleles if frequencies exceeded 50%. The 5′- and 3′-end sequences were determined by Rapid Amplification of cDNA Ends as described previously [12]. Sequences were analyzed and assembled by using Geneious software (Biomatters, Auckland, New Zealand). Nucleotide sequences for 15-I-752 (LC212972) and 15-I-1105 (LC212973) were deposited in the GenBank/EMBL/DDBJ databases.

**Fig. 2:**
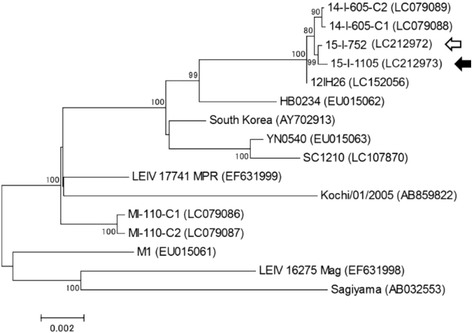
Phylogenetic analysis of complete Getah virus genome sequences. The Getah virus strain isolated from a pig (15-I-1105) is indicated by the black arrow. The strain isolated from a horse during the 2015 outbreak (15-I-752) is indicated by the white arrow. The percent bootstrap support is indicated by the values at each node; values less than 70% were omitted. MI-110-C1, MI-110-C2, 14-I-605-C1, and 14-I-605-C2 are Getah virus isolates from horses; 12IH26, HB0234, YN0540, SC1210, LEIV17741 MPR, M1, LEIV16275 Mag, and Sagiyama are isolates from mosquitoes; South Korea and Kochi/01/2005 are isolates from pigs

Phylogenetic analysis of nucleic acid sequences was performed with MEGA 7 software [13]. A phylogenetic tree based on complete genome sequences was constructed by using the maximum-likelihood method, and statistical analysis was performed by using bootstrap tests (1000 replicates). Nucleotide sequence accession numbers for the Getah virus strains used in the phylogenetic analysis were as follows: MI-110-C1 (LC079086), MI-110-C2 (LC079087), 14-I-605-C1 (LC079088), and 14-I-605-C2 (LC079089) were isolated from horses; HB0234 (EU015062), YN0540 (EU015063), SC1210 (LC107870), LEIV 17741 MPR (EF631999), M1 (EU015061), LEIV 16275 Mag (EF631998), and Sagiyama (AB032553) were isolated from mosquitoes; and South Korea (AY702913) and Kochi/01/2005 (AB859822) were isolated from pigs (Fig. 2).

## Results

### Rates of seropositivity to Getah virus among pigs in south Ibaraki and north Chiba

The rates of seropositivity to Getah virus in autumn 2012–2015 in south Ibaraki and 2010–2015 in north Chiba are summarized in Tables 1 and 2. For those serum batches that contained at least one seropositive serum, the exact sampling date, the location, and the numbers of positive/tested samples are summarized in Tables 3 and 4. Seropositivity rates in 2012–2013 in south Ibaraki and 2010–2012 in north Chiba ranged from 0% to 1.6% (Tables 1 and 2). In these years, each positive serum sample was the only one in each batch, and no batches had multiple seropositive samples (Tables 3 and 4). In contrast, seropositivity rates in 2014 and 2015 in south Ibaraki were significantly higher than those in the previous years: 28.8% in 2014 (*P* < 0.01) and 65.0% in 2015 (*P* < 0.01). The proportions of batches that had positive sera were also high (4/5 in 2014 and 7/7 in 2015; Table 1). Among the 11 batches that were positive in 2014–2015 in south Ibaraki, nine had multiple seropositive samples with VN antibodies (Table 3). Seropositivity rates in north Chiba from 2013 to 2015 were significantly higher than those in the previous years: 14.1% in 2013 (*P* < 0.01), 17.8% in 2014 (*P* < 0.01), and 48.0% in 2015 (*P* < 0.01). In 2013, 6/27 batches had positive sera; this value was 3/9 in 2014 and 5/5 in 2015 (Table 2). Eleven out of 14 seropositive batches in 2013–2015 in north Chiba included multiple seropositive samples (Table 4).

**Table 3:** Serum batches that were collected in south Ibaraki in autumn 2012–2015 and that included VN antibody-positive pigs

Year	Date	City	Virus-neutralization test (positive/tested samples)	RT-PCR (positive/tested pools)
2012	Nov 8	Tsuchiura	1/17	ND
2014	Sep 8	Inashiki	6/20	2/2
	Oct 20	Inashiki	2/6	0/1
	Oct 31	Inashiki	10/15	0/1
	Nov 25	Ushiku	1/15	ND
2015	Sep 28	Tsuchiura	2/6	1/1^a^
	Oct 1	Tsuchiura	46/52	0/1
	Oct 1	Tsuchiura	7/31	0/4
	Nov 10	Inashiki	14/14	ND
	Nov 17	Tsuchiura	5/5	ND
	Nov 27	Tsuchiura	1/7	ND
	Dec 15	Tsuchiura	2/2	ND

ND, not done

^a^Getah virus was isolated

**Table 4:** Serum batches that were collected in north Chiba in autumn 2010–2015 and that included VN antibody-positive pigs

Year	Date	City/Town	Virus-neutralization test (positive/tested samples)	RT-PCR (positive/tested pools)
2010	Sep 1	Katori	1/5	ND
	Sep 14	Katori	1/4	ND
2013	Oct 18	Tako	1/5	0/1
	Oct 24	Katori	3/5	0/1
	Oct 31	Katori	2/5	0/1
	Nov 19	Tohnosho	5/5	ND
	Nov 21	Katori	4/5	ND
	Dec 13	Katori	4/5	ND
2014	Oct 1	Tomisato	3/5	0/1
	Oct 1	Tomisato	2/5	0/1
	Oct 1	Narita	3/5	0/1
2015	Oct 23	Tomisato	1/10	1/2
	Oct 27	Narita	1/10	0/2
	Oct 29	Katori	9/10	0/1
	Oct 29	Sosa	9/10	0/1
	Oct 29	Sosa	4/10	0/2

ND, not done

### Detection of viral RNA in pig sera and virus isolation

For efficient isolation of Getah virus, we first performed RT-PCR to detect Getah virus RNA in pooled sera. Because the epidemic season of Getah virus is generally from September to October, we set pools from the batches collected in these two months of each year and that included seropositive samples. Five sera from each batch were pooled (*n* = 24), and each pool included as many seronegative samples as possible. This was because virus recovery might not have been achieved from antibody-positive sera: the presence of antibodies should indicate that the viremic phase has finished. Getah virus RNA was detected in four out of 24 pools: two pools collected on 8 September 2014 in Inashiki city (Ibaraki Pref.), one pool collected on 28 September 2015 in Tsuchiura city (Ibaraki Pref.), and one pool collected on 23 October 2015 in Tomisato town (Chiba Pref.) (Tables 3 and 4).

**Fig. 3:**
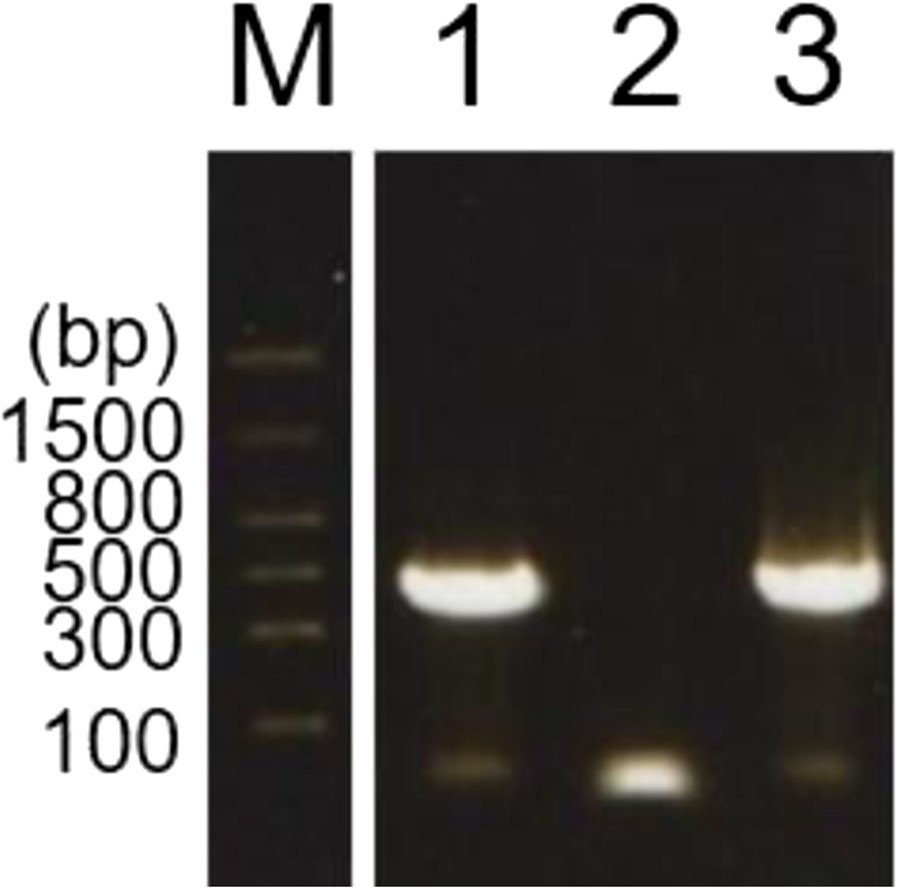
Detection of viral RNA by RT-PCR for Getah virus from the supernatant of Vero cells inoculated with a pig serum. After virus isolation in Vero cells, one serum collected on 28 September 2015 in Tsuchiura city (Ibaraki Pref.) showed cytopathic effects; the nucleic acid was extracted from the culture supernatant, and RT-PCR for the *nsP1* gene was performed. M, marker; lane 1, supernatant of the cytopathic effects-positive culture; lane 2, negative control; lane 3, positive control

The sera included in the Getah virus RNA-positive pools were inoculated with Vero cells for virus isolation. Out of 20 sera from four pools, one serum collected on 28 September 2015 in Tsuchiura city (Ibaraki Pref.) showed cytopathic effects; the strain isolated was confirmed as Getah virus by RT-PCR for the *nsP1* gene with the predicted length of 434 base pairs (Fig. 3) and sequencing; it was designated strain 15-I-1105.

### Complete genome sequencing of Getah virus strains isolated from pig and horses

To clarify the interactions between the Getah virus strains isolated from the pig and the horses, we compared the complete genome sequence of strain 15-I-1105 with those of other strains, including the ones isolated during the outbreaks in horses. Phylogenetic analysis showed that strain 15-I-1105 was located in the same cluster as the strains isolated from horses during the 2014 outbreak (14-I-605-C1, 14-I-605-C2), one strain isolated from a horse during the 2015 outbreak (15-I-752), and one strain isolated from mosquitoes in Nagasaki Prefecture in 2012 (12IH26, [14]) (Fig. 2). Strain 15-I-1105 had extremely high nucleotide sequence identity to each strain in the same cluster, with 99.90% to 14-I-605-C1, 99.89% to 14-I-605-C2, 99.94% to 15-I-752, and 99.91% to 12IH26. Small numbers of amino acid differences between 15-I-1105 strain and the strains from horses were found mostly in the nsP1–2–3-4 polyprotein (Table 5). 15-I-1105 strain had different amino acids in five sites against 15-I-752 strain, and seven sites against both 14-I-605-C1 and 14-I-605-C2 strains (Table 5).

**Table 5:** Amino acid differences between the strains from the pig and horses

		Pig strain	Horse strains		
Protein	Position	15-I-1105	14-I-605-C1	14-I-605-C2	15-I-752
Non-structural polyprotein^a^					
nsP1	58	I	V	V	V
	354	A	A	V	A
	385	V	L	V	V
	427	I	T	T	I
	475	I	M	M	M
nsP3	1694	A	A	A	V
	1814	V	E	E	E
nsP4	2114	T	A	A	T
Structural polyprotein^b^					
E1	1113	S	N	N	N

^a^Non-structural polyprotein (nsP1–2–3-4) has 2467 amino acids

^b^Structural polyprotein (C-E3-E2-6 K-E1) has 1253 amino acids

## Discussion

Pigs are regarded as natural hosts and amplifiers of Getah virus because of the high virus titers found in the viremic phase after experimental infection and the high seroprevalence among pigs in the field [4, 15, 16]. As described above, Ibaraki and Chiba prefectures, where our survey areas were located, are major producers of pigs in Japan. Unlike in Japan’s other pig production areas, in south Ibaraki and north Chiba many racehorse farms and piggeries are located close together. The numbers of horse farms in which racehorses stayed before introduction to the Miho training center in 2015 were 20 in south Ibaraki and 11 in north Chiba. The existence of mosquito vectors of Getah virus has been confirmed in these areas [7]. Therefore, it is likely that transmission of Getah virus between pigs and horses can occur easily there.

As we showed here, Getah virus was epizootic among pigs in south Ibaraki and north Chiba in autumn 2014 and 2015; this was consistent with the epizootic periods among racehorses [8, 9]. The number of affected horses at the Miho training center was 33 in 2014 and 30 in 2015, and the maximum seropositivity rates among horses that were introduced from farms in Ibaraki and Chiba to the center were 42.9% in 2014 and 34.9% in 2015 [8–10]. Although we tested a portion of the pig farms in these areas, seropositive batches were found at multiple sites during these epizootic seasons. In most of the seropositive batches, multiple seropositive samples were found at each site. We therefore considered that individual seropositive pigs were not infected sporadically, but that the virus was circulating in the pig population in these areas. However, to our knowledge, there have been no published reports of an increased incidence of disease potentially caused by Getah virus in local pigs. Despite the viral spread, most of the infections in pigs seemed to be sub-clinical or not reported.

In north Chiba, a high seropositivity rate was found in pigs not only in 2014 and 2015 but also in 2013, when there was no detectable outbreak in horses. In our previous study we investigated seropositivity rates among racehorses introduced from Ibaraki and Chiba prefectures to the training center; the maximum seropositivity rate from June to October 2013 was 2.0% [10]. In that study, of 250 horse sera examined over a total of five months, 62 were from horses introduced from Chiba, and all of them were seronegative for Getah virus. Therefore, it seemed that virus circulation in north Chiba in 2013 occurred only among pigs, and not among horses in the area. The absence of a detectable outbreak in horses in 2013 might be attributable to the fact that virus circulation among pigs did not occur in south Ibaraki, which contains the Miho training center and greater numbers of horse farms than are found in north Chiba.

Genomic comparison revealed that the strain isolated from a pig in 2015 (15-I-1105) was closely related to the strains isolated from horses in 2014 (14-I-605-C1 and 14-I-605-C2) and 2015 (15-I-752), with extremely high nucleotide sequence identities. This finding, together with the serological data, indicates this was the first convincing case that Getah virus circulation among horses and pigs occurred interactively in a specific area. On the basis of the theory that pigs are natural hosts of Getah virus, the outbreak among racehorses in 2014 and 2015 was likely preceded by viral amplification in pigs. However, the virus might not be transferred in a one-way manner from pigs to horses; instead, it might circulate interactively between the two species, because the high-level viremia required for vector transmission has also been observed in infected horses [5]. Therefore, we could not conclude that the epizootics in 2014 and 2015 were initiated from infected pigs, and it was also possible that the virus was derived from infected horses that were transferred from other epizootic areas. Because horses are moved from one farm to the other farms frequently, such movement in the area should be responsible for virus transfer among horse population. However, animal and human movements between horse farms and pig farms are considered to be rarely occurred, and may not be the major route of viral transfer between the two species. As we described above, the pig farms and horse farms are closely located in the area, and some of them were located within several hundred meters distance. In this regard, the virus should be transferred by flight activities of infected mosquitoes.

Our phylogenetic analysis of various Getah virus strains revealed that the strain from a pig (15-I-1105) and strains from recent outbreaks in horses (14-I-605-C1, 14-I-605-C2, and 15-I-752) formed a cluster with a strain isolated from mosquitoes in Nagasaki Prefecture in 2012 (12IH26, [14]) (Fig. 2). Phylogenetic analyses by Kobayashi et al. using the sequences for the *E2*, *capsid*, and *nsP1* genes suggested that this cluster containing 12IH26, 14-I-605-C1, and 14-I-605-C2 was closely related to the recent Chinese and South Korean strains but only distantly related to Japanese strains from the 1970s and 1980s [14]. Although it is still unclear how and when the virus was brought into Japan from overseas and spread into Ibaraki and Chiba prefectures, this cluster of viruses seems to have been established in these areas and was circulating among both pigs and horses in recent years.

## Conclusions

Serological investigation of Getah virus in pigs revealed that the virus was circulating in south Ibaraki and north Chiba in 2014 and 2015, during the same period as outbreaks in racehorses. The Getah virus strain isolated from an infected pig was closely related to those from horses during that period. This was the first convincing case of interactive circulation of Getah virus among both pigs and horses in a specific area. Consistent with the findings of previous studies, our current data suggest that pigs are important natural hosts and amplifiers. Periodic surveillance of pigs over a larger area would be useful for estimating the risk of epizootics in horses.

## Abbreviations

MEM: minimum essential medium
nsP1: non-structural protein 1
VN: virus-neutralization
